# A Novel Sterol Regulatory Element-Binding Protein Gene (*sreA*) Identified in *Penicillium* *digitatum* Is Required for Prochloraz Resistance, Full Virulence and *erg11* (*cyp51*) Regulation

**DOI:** 10.1371/journal.pone.0117115

**Published:** 2015-02-20

**Authors:** Jing Liu, Yongze Yuan, Zhi Wu, Na Li, Yuanlei Chen, Tingting Qin, Hui Geng, Li Xiong, Deli Liu

**Affiliations:** Hubei Key Laboratory of Genetic Regulation and Integrative Biology, School of Life Science, Central China Normal University, Wuhan, 430079, China; Geisel School of Medicine at Dartmouth, UNITED STATES

## Abstract

*Penicillium* *digitatum* is the most destructive postharvest pathogen of citrus fruits, causing fruit decay and economic loss. Additionally, control of the disease is further complicated by the emergence of drug-resistant strains due to the extensive use of triazole antifungal drugs. In this work, an orthologus gene encoding a putative sterol regulatory element-binding protein (SREBP) was identified in the genome of *P*. *digitatum* and named *sreA*. The putative SreA protein contains a conserved domain of unknown function (DUF2014) at its carboxyl terminus and a helix-loop-helix (HLH) leucine zipper DNA binding domain at its amino terminus, domains that are functionally associated with SREBP transcription factors. The deletion of *sreA* (ΔsreA) in a prochloraz-resistant strain (PdHS-F6) by *Agrobacterium* *tumefaciens*-mediated transformation led to increased susceptibility to prochloraz and a significantly lower EC_50_ value compared with the HS-F6 wild-type or complementation strain (CO*sreA*). A virulence assay showed that the Δ*sreA* strain was defective in virulence towards citrus fruits, while the complementation of *sreA* could restore the virulence to a large extent. Further analysis by quantitative real-time PCR demonstrated that prochloraz-induced expression of *cyp51A* and *cyp51B* in PdHS-F6 was completely abolished in the Δ*sreA* strain. These results demonstrate that *sreA* is a critical transcription factor gene required for prochloraz resistance and full virulence in *P*. *digitatum* and is involved in the regulation of *cyp51* expression.

## Introduction

Fungal infection is one of the three main diseases of crops, other than bacteria and viruses that can result in reductions in agricultural output [1]. Green mold caused by the ascomycete fungi *P*. *digitatum* is the most destructive disease of citrus fruit, responsible for up to 90% of total crop losses during postharvest packing, storage, transportation, and marketing [2]. Control of *P*. *digitatum* is critical to solving this worldwide problem; however, the emergence of drug-resistant strains due to excessive use of demethylation inhibitor (DMI) fungicides has resulted in less efficient control of this disease [3–5]. Under this circumstance, an understanding of the potential molecular mechanisms involved in DMI resistance is of great significant because it will provide a basis for the designing of novel antifungal chemicals with greater efficacy.

Fungal resistance to azole reagents has been attributed variously to genetic mutations in its target *erg11* (*cyp51*), and/or the upregulation of efflux pump genes such as *MDR1*, *CDR1*, and *CDR2* [6]. Filamentous fungi, particularly *Ascomycetes*, often possess two or more CYP51 paralogous: in *Aspergillus fumigatus* (two), *A*. *nidulans* (two), *A*. *flavus* (three), *Magnaporthe oryzae* (two) and species of *Fusarium*, including *F*. *verticillioides*, *F*. *oxysporum* f. sp. *lycopersici* and *F*. *graminearum* (three) [7]. Three sterol 14α-demethylase (CYP51) genes were found in *P*. *digitatum*, and evidence on the transcriptional regulation of these target genes has emerged to explain the drug-resistant mechanisms of *P*. *digitatum* [8]. Hamamoto *et al*. [9] reported that duplication of a 126-bp DNA element in the *cyp51* promoter region led to the increasing resistance of *P*. *digitatum* strains to the antifungal drug imazalil. Another case of imazalil-resistance is associated with up-regulated CYP51 expression caused by the insertion of a 199-bp miniature inverted-repeat transposable element (MITE) in the promoter region [10]. In addition to the overexpression of the *cyp51*, transporter genes from the ATP-binding cassette (ABC) transporter family and the major facilitator super family (MFS) have also been associated with fungicide resistance in *P*. *digitatum*. As reported, seven ABC proteins induced by imazalil in *P*. *digitatum* contributed to DMI fungicide efflux, and *PdMFS1*, a typical MFS member, is involved in imazalil-resistance and pathogenicity of *P*. *digitatum* [11–14].

The drug resistance mechanisms of fungi may rely on transcription factors acting on effector genes that have been characterized in a number of clinical species [15]. CaUpc2 is a well-characterized transcription factor in *Candida albicans* that is associated with drug resistance and sterol metabolism. CaUpc2 is required for induction of the *erg2* and *erg11* ergosterol biosynthesis genes. *CaUpc2* deletion strains exhibit reduced ergosterol levels and no induced expression of *cyp51* orthologs, which may explain the increased susceptibilities of these strains [16–17]. It was also reported that gain-of-function mutations in *CaUpc2* could contribute to azole resistance [18–19]. However, orthologs of *upc2* do not appear to exist in *P*. *digitatum*, thus it is possible that other transcription factors in *P*. *digitatum* serve similar functions as Upc2 in *C*. *albicans*.

Sterol regulatory element-binding proteins (SREBPs) contain a basic helix-loop-helix domain with a specific tyrosine residue and function as membrane-bound transcription factors required for virulence, resistance to antifungal drugs, and hypoxia responses in fungi [20]. Sre1, an SREBP transcription factor first characterized in the fission yeast *Schizosaccharomyces pombe*, is required for adaptation to hypoxia and anaerobic conditions [21]. Sre1, an SrbA ortholog identified in *A*. *fumigates*, a well investigated opportunistic pathogenic mold, is not only required for hypoxia response, cell polarity, and full virulence, but also regulates resistance to the azole antifungal drugs [22–24]. A null mutant of SrbA was unable to grow under hypoxia and displayed increased susceptibility to the azole antifungal drugs, demonstrating that SrbA mediate triazole susceptibility through the direct regulation of *erg11A* expression [24]. Although Upc2 is not an ortholog of SREBPs, these two classes of transcription factors have analogous functions, similar localization and activation patterns, and are proposed to be an example of convergent evolution in the fungal kingdom [24]. Based on these reports, we deduced that *P*. *digitatum* might also have a SREBP-like transcript factor involved in antifungal drug responses.

Prochloraz is a type of triazole fungicide that is widely used in Europe, Australia, Asia and South America for gardening and agriculture [25]. However, little is known about prochloraz resistance mechanisms of in *P*. *digitatum*. In this study, we report the identification and characterization of an ortholog of *Aspergillus* SrbA, SreA, in *P*. *digitatum*. By constructing an *sreA*-disrupted strain and a complemented strain, we analyzed the effects of SreA on full virulence, prochloraz (PRC) resistance and ergosterol biosynthetic genes. Our results provide further insight into the molecular mechanisms of fungicide resistance in *P*. *digitatum*.

## Materials and Methods

### Strains and media

The *P*. *digitatum* strain HS-F6 previously isolated by our research group [26] was used in this study. All mutant strains were generated from PdHS-F6 through *A*. *tumefaciens*-mediated transformation. Conidial suspensions of wild-type HS-F6 and the mutant strains were stored in 20% glycerol solution at -80°C. PdHS-F6 is highly resistant to triazole drug prochloraz with an EC_50_ value of 7.896 mg/l. *P*. *digitatum* strains were cultured on potato dextrose agar (PDA) medium (extract of 200 g potato boiled water, 20 g dextrose, and 15 g agar per liter) at 25°C. The mycelium used for DNA and RNA extraction was obtained by inoculating 20 μl of a conidial suspension (10^6^ spores ml^-1^) into 100 ml liquid potato dextrose medium (PDA without agar) and growing on a rotary shaker (160 rpm) at 25°C for three days. The *A*. *tumefaciens* EHA105 strain, which was generously provided by Dr. Daohong Jiang (Huazhong Agricultural University, China), was grown in YEP medium [26], minimal medium (MM) (K_2_HPO_4_ 2 g/l, KH_2_PO_4_ 1.45 g/l, MgSO_4_·7H_2_O 0.6 g/l. NaCl 0.3 g/l, CaCl_2_·2H_2_O 0.01 g/l, glucose 2 g/l, FeSO_4_ 0.001 g/l, ZnSO_4_·7H_2_O 0.005 g/l, CuSO_4_·5H_2_O 0.005 g/l, H_3_BO_3_ 0.005 g/l, MnSO_4_·H_2_O 0.005 g/l, Na_2_MoO_4_·2H_2_O 0.005 g/l, NH_4_NO_3_ 0.5 g/l) and induction medium (IM) (MM salts with 40 mM 2-[N-morpholino] ethanesulfonic acid (MES) pH 5.3, 10 mM glucose, 0.5% (v/v) glycerol) supplemented with 10 μg/ml kanamycin and 60 μg/ml rifampicin at 28°C.

### Cloning and sequencing of *sreA* from *P*. *digitatum*

Based on the DNA sequence of *A*. *fumigatus SrbA* (GenBank accession no.XM_744169), we identified an SREBP protein-encoding gene *Pc20g05880* (GenBank accession no.XM_002563071) in *P*. *chrysogenum*. Given that the genome of *P*. *digitatum* and *P*. *chrysogenum* share high similarity [27], two pairs of specific primers *sreA-a*, *sreA-b*, *sreA-c* and *sreA-d* (**Table 1**) were designed according to the relatively conserved sequences of *A*. *fumigatus sreA* and *P*. *chrysogenum Pc20g05880* after sequence alignment with ClustalW. Two approximately 1000-bp DNA fragments were amplified from genomic DNA of *P*. *digitatum* by PCR, and then cloned into pMD18-T vector (TaKaRa Biotech. Co., Dalian, China) for sequencing. Then an approximately 2000-bp DNA fragment of *P*. *digitatum sreA* was obtained after sequence-assembling. The 5’ flanking DNA sequence of *sreA* was amplified by genome walking using the Genome Walking Kit (TaKaRa Biotech. Co., Dalian, China) with specific primers *sreA-e*, *sreA-f and sreA-g* (**Table 1**). The 3’ flanking unknown DNA sequence of *sreA* was amplified using SMATer RACE 5’/3’ Kit (TaKaRa Biotech. Co., Dalian, China) with specific primers *sreA-h* and *sreA-i*. The DNA sequence of *sreA* has been deposited in GenBank under accession number KJ939329.

**Table 1:** Primers used in this study.

Name	Sequence(5’-3’)	Purpose
sreA-a	ATTTGAACTACAAAGACTTCTC	PCR primers used to amplify the DNA fragment of *sreA*.
sreA-b	TACCACTCTCGGAAGAACCTATG	PCR primers used to amplify the DNA fragment of *sreA*.
sreA-c	GCCGGTCTGATGGTTCTTGAAGG	PCR primers used to amplify the DNA fragment of *sreA*.
sreA-d	TCCAATGAGAGAAAGCTGGACTGG	PCR primers used to amplify the DNA fragment of *sreA*.
sreA-e	ACACGAGGCCTAAGTTTTGTTGCTG	PCR primers used to amplify the 5’ unknown DNA sequence of *sreA*.
sreA-f	ATCAAGCCGACCGGAAGATTAGGC	PCR primers used to amplify the 5’ unknown DNA sequence of *sreA*.
sreA-g	TTGATCTCCTTCCGAGAACATGGGC	PCR primers used to amplify the 5’ unknown DNA sequence of *sreA*.
sreA-h	ATACTGGGCCCGAAATGCCTACACC	PCR primers used to amplify the 3’ unknown DNA sequence of *sreA*.
sreA-i	TAAGAAATACAGGACCCGTCGACGC	PCR primers used to amplify the 3’ unknown DNA sequence of *sreA*.
sreA-1	CCCTCGAGATGTCTGGCCCCAATATGGAG	PCR primers used to amplify 5’ fragments of *sreA*.
sreA-2	GGACTAGTCAGCAAAGACTCCATGGTTTGC	PCR primers used to amplify 5’ fragments of *sreA*.
sreA-3	CGAGCTCTTTCGAAGCAAGCGAGAAGG	PCR primers used to amplify 3’ fragments of *sreA*.
sreA-4	GGGGTACCTCAGGCAGGGACATTTTGCA	PCR primers used to amplify 3’ fragments of *sreA*.
sreA-F	ATGGATGTCTGGCCCCAATATGGAG	PCR primers used to amplify the ORF of *sreA* gene.
sreA-R	TCAGGCAGGGACATTTTGCAC	PCR primers used to amplify the ORF of *sreA* gene.
sreA-F1	GGACTAGTGGCAATAGTGGAGACTA GCAC	PCR primers used to amplify *sreA*, including its promoter and terminator.
sreA-R1	GGACTAGTCTGATAACATTCCATTTCCC	PCR primers used to amplify *sreA*, including its promoter and terminator.
cyp51A-F	CACTGGATTCCTTTCATTGGG	PCR primers used to amplify *cyp51A* gene by quantitative real-time PCR.
cyp51A-R	TCCGAAGACGGGGGTTGTAA	PCR primers used to amplify *cyp51A* gene by quantitative real-time PCR.
cyp51B-F	GAGTTCATCCTCAATGGCAAGC	PCR primers used to amplify *cyp51B* gene by quantitative real-time PCR.
cyp51B-R	CTTAGAGTTGGGGCAATCGTAGAC	PCR primers used to amplify *cyp51B* gene by quantitative real-time PCR.
cyp51C-F	TGTTCAAGCAGCCATTCAAGC	PCR primers used to amplify *cyp51C* gene by quantitative real-time PCR.
cyp51C-R	CAAGTTGGGTCCGACGAAATA	PCR primers used to amplify *cyp51C* gene by quantitative real-time PCR.
β-actin-F	TGTCACCAACTGGGACGATA	PCR primers used to amplify *β-actin* gene by quantitative real-time PCR.
β-actin-R	GAGCTTCGGTCAAGAGGATG	PCR primers used to amplify *β-actin* gene by quantitative real-time PCR.

Note: The underlined sequences represent different restriction enzyme sites.

### DNA and protein analysis

The DNA sequence of *sreA* was analyzed using NCBI BLAST and BioEdit software. A protein analysis was performed using the NCBI BLAST and Interpro (http://www.ebi.ac.uk/interpro/) programs. TMHMM software (version 2.0) was used to predict the transmembrane domains of SreA.

### Construction of an *sreA* disruption plasmid

**Fig 1:**
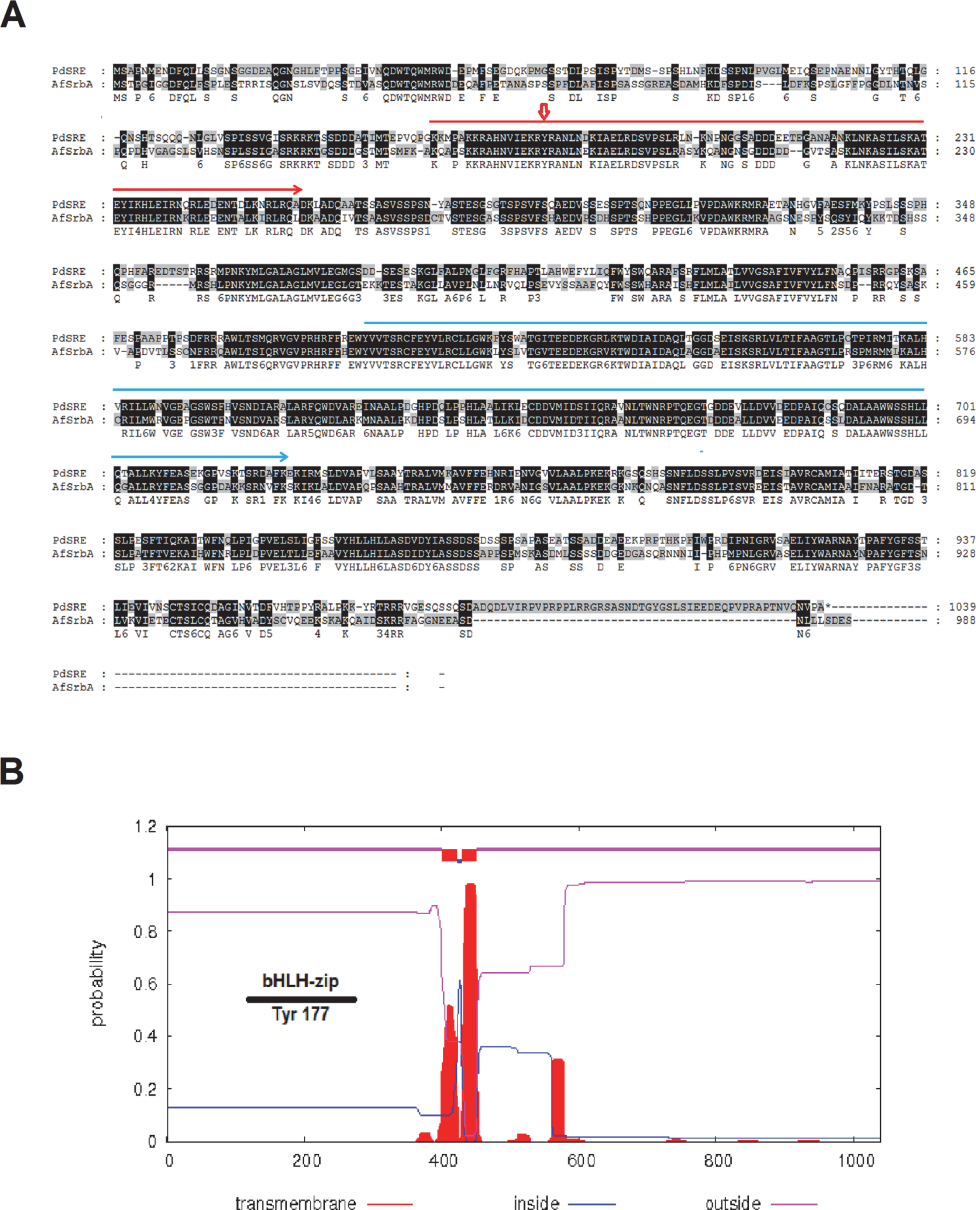
Sequence analysis of *sreA* from PdHS-F6. A: Homology between the amino acid sequences of SreA from *P*. *digitatum* and SrbA in *A*. *fumigatus*. The sequence under the red line indicates basic helix-loop-helix-leucine zipper (bHLH-zip) domain located at the N-terminus of the protein. The sequence under the blue line indicates the DUF2014 domain specific to the ER membrane-bound transcription factor Sterol Regulatory Element Binding Proteins (SREBPs). The arrow indicates the amino acid position of the highly conserved tyrosine in the bHLH zipper domain (bHLH-zip). B: Transmembrane domain prediction plots of SreA.

The plasmid pTFCM containing the *PtrpC* promoter and *TtrpC* terminator from *A*. *nidulans* and carrying the *hph* gene which confers resistance to hygromycin B as selective marker was generously provided by Dr. Daohong Jiang (Huazhong Agricultural University, China). The *sreA* disruption plasmid was constructed by inserting the up-stream and down-stream flanking sequences of *sreA* into the pTFCM vector [28]. To this end, a 1-kb DNA fragment of the *sreA* 5’ coding sequence was amplified from *P*. *digitatum* genomic DNA using primers *sreA-1* and *sreA-2* (**Table 1**) and cloned into pMD18-T vector. After sequencing, the fragment containing *sreA* 5’ coding sequence was digested by *Spe*I and *Xho*I and sub-cloned into pTFCM between *Spe*I and *Xho*I sites to generate the pTFCM-L plasmid. Next, another 1-kb fragment containing the *sreA* 3’ coding sequence was amplified and cloned into the pMD18-T vector for sequencing using primers *sreA-3* and *sreA-4* (**Table 1**). After digestion by restriction enzymes *Kpn*I and *Sac*I, the fragment containing *sreA* 3’ coding sequence was inserted between the *Kpn*I and *Sac*I sites of pTFCM-L to generate the *sreA* disruption plasmid pTFCM-L-R (**Fig. 1A**).

### Construction of *sreA* complementation plasmid

The *sreA* complementation plasmid was obtained by a PCR strategy (**Fig. 1A**). A DNA fragment containing the *sreA* open reading frame as well as its promoter and terminator (4385bp) was amplified using genomic DNA of *P*. *digitatum* as a template and *sreA-F1*/*sreA-R1* as primers (**Table 1**). The PCR conditions were as follows: 3 min at 94°C followed by 30 cycles each of 30 s at 94°C, 1 min at 60°C, and 2 min at 72°C. A GeneAmp 9700 thermal cycler (Perkin-Elmer [PE] Applied Biosystems, Foster City, Calif., USA) was used with LA Taq polymerase (TaKaRa Biotech. Co., Dalian, China). The amplified fragment was digested by *Spe*I and cloned into plasmid pTFCM*-neo* to obtain the complementation plasmid pTFCM*-neo-sreA*. The pTFCM*-neo* plasmid was constructed by replacing the *hph* cassette between two *Xba*I sites in pTFCM with a *neo* cassette that confers resistance to the antibiotic G418.

### Transformation of *P*. *digitatum*

Prior to the transformation, the recombinant plasmids pTFCM-L-R and pTFCM-*neo*-*sreA* were transformed into *A*. *tumefaciens* strain EHA105 by a heat-shock method [29]. Next, *A*. *tumefaciens*-mediated transformation was performed to obtain *sreA* disruption (Δ*sreA*) and complementation (CO*sreA*) strains [28, 30]. First, *A*. *tumefaciens* strains harboring the disruption plasmids were recovered on YEP plates at 28°C for two days. A single colony containing pTFCM-L-R plasmids was selected and cultured in MM medium before transfer into IM medium cointaining 200 μM acetosyringone; the cell density was adjusted to *OD*_600_ = 0.15. After incubating at 28°C with shaking at 180 rpm for 6 h, equal volumes of *A*. *tumefaciens* culture and *P*. *digitatum* conidial suspensions (10^6^ spores ml^-1^) were mixed and transferred to a lens paper on an IM agar plate for cultivation at 25°C for 3 days. Finally, the lens paper was transferred onto the PDA medium containing 50 μg/ml hygromycin B and 50 μg/ml cefoxitin to select for *sreA* disruption strains (Δ*sreA*). To obtain *sreA* complementation strains (CO*sreA*), one selected Δ*sreA* strain was transformed with the complementation plasmid pTFCM*-neo-sreA* according to the protocol of Δ*sreA* strain construction, with the modification that G418 (200 μg/ml) was used to select transformants instead of hygromycin B. The Δ*sreA* and CO*sreA* transformants were confirmed by PCR using primers *sreA-F* and *sreA-R* (**Table 1**) and a Southern blot analysis.

### Southern blot analysis

Genomic DNA was extracted from the HS-F6 wild-type, Δ*sreA*, and CO*sreA* strains using Biospin Fungus DNA Extraction Kit (BioFlux, Tokyo, Japan). Approximately 30 μg genomic DNA was digested with *Hind*III at 37°C for 12 hours, electrophoresed on a 1% agarose gel and transferred to a positively charged nylon membrane. A 1003-bp digoxigenin-labeled probe specific to the 5’ region of *sreA* was generated using the DNA Probe Labeling Kit (TIANDZ, Beijing, China) by PCR with primers *sreA-1* and *sreA-2* (**Table 1**) and hybridized with the genomic DNA blot. DIG Random Labeling and Detection Kit II (BOSTER, Wuhan, China) was used for color detection following the manufacturer’s protocol.

### Assays of vegetative growth and prochloraz EC_50_

To compare the growth of HS-F6 wild-type, Δ*sreA*, and CO*sreA* strains, a 0.8-mm mycelial plug was obtained from a PDA plate with 50 μl of the corresponding conidial suspension (1×10^6^ spores ml^-1^) coated on the surface and then cultured on a new PDA plate. After four days’ cultivation at 25°C, the diameters of different colonies were measured.

EC_50_ values of prochloraz (1-{N-propyl-N-[2-(2,4,6-trichlorophenoxy)ethyl]carbamoyl}; PRC) for the different strains were measured according to [31] with modifications. Briefly, 50 μl of a conidial conidia suspension (10^6^ spores ml^-1^) of the HS-F6 wild-type and mutant strains was coated onto a PDA plate respectively and cultivated at 25°C for 24 h. Mycelial plugs (approximately 0.8 mm diameter) were then obtained from the plate using a punch and placed on the center of PDA plates containing different concentrations of prochloraz. After incubation at 25°C for 6 days, the diameters of the colonies were measured. Three replicates were used for each experiment. The average of the colony diameters in each independent test was used for EC_50_ calculation by software SPSS 10.0.

### Virulence assay

Mature citrus fruits (*Citrus sinensis*) were purchased from a fruit market in Hongshan district, Wuhan. The fruits were washed with distilled water and dried at room temperature before inoculation. Virulence assays for the HS-F6 and mutant strains were performed directly on citrus fruits. Firstly, a 2-mm deep hole was made on the pericarp using a 1-ml pipett tip. Then 3 μl of a conidial suspension (10^6^ spores ml^-1^) of the HS-F6 wild-type or mutants was injected into the hole. After incubation at 25°C for three days, the diameters of the disease spots formed were measured and compared.

### RNA extraction and quantitative real-time PCR (qRT-PCR)

qRT-PCR was used to analyze *cyp51* gene expression. Before RNA extraction, 20 μl of a conidial suspension (10^6^ spores ml^-1^) of *P*. *digitatum* HS-F6 and Δ*sreA* strains was cultured in PDB medium at 25°C for 72 h. In the prochloraz-treatment experiment, 7 μg/ml prochloraz (about the concentration of EC_50_) was added to the PDB medium with shaking for an extra 6 h after cultured at 25°C for 48 h. The mycelia were then filtered and washed several times using double distilled water. Total RNA was extracted using RNAiso Plus (TaKaRa Biotech. Co., Dalian, China) according to the manufacturer’s protocol. All RNA samples were treated with DNase I (TaKaRa Biotech. Co., Dalian, China). First-strand cDNA was prepared using All-in-one First strand cDNA Synthesis Kit (Genecopoeia, Guangzhou, China) following the manufacturer’s protocol. qRT-PCR was performed using a BIO-RAD CFX96 q-PCR system with SYBR Green I fluorescent dye detection. The mRNA abundance was normalized using the housekeeping gene *β-actin*, and the relative expression levels were calculated using the 2^-ΔΔCt^ method [32]. The primers used to amplify *cyp51A/B/C* and *β-actin* genes in qRT-PCR are listed in **Table 1**.

### Statistical analysis

All results presented with statistical significance were analyzed with an unpaired two-tailed Student’s t test and two-way ANOVA. *P*<0.05 was considered significant.

## Results

### Cloning and sequence analysis of *sreA* from PdHS-F6

A 3120-bp DNA fragment of *sreA* ORF was amplified from PdHS-F6 genomic DNA using primers *sreA-F* and *sreA-R* (**Table 1**), sequenced, and then analyzed by NCBI BLAST. The *sreA* ORF is predicted to encode a protein of 1040 amino acids that is a putative HLH transcription factor and an ortholog of *A*. *fumigatus* transcription factor SrbA. The nucleotide homology between *sreA* and *srbA* is 63%; protein homology is 61% (**Fig. 1**). The nucleotide homology between *sreA* and *P*. *chrysogenum Pc20g05880* is 89%.

Two highly conserved domains in SreA were identified. A basic helix-loop-helix-leucine zipper (bHLH-zip) domain located in the N-terminus of the protein contains a unique tyrosine residue (at site 177) that distinguishes SREBPs from other bHLH transcriptional factors [33]; a domain DUF2014 of unknown function is located in the C-terminus of the protein. This domain is found at the C-terminus of a family of ER membrane bound transcription factors called sterol regulatory element binding proteins (SREBP). SreA is predicted to contain two transmembrane domains, suggesting that this protein is a membrane-bound transcriptional factor with a topology similar to vertebrate SREBPs.

### Construction of *sreA*-disruption and -complementation strains

**Fig 2:**
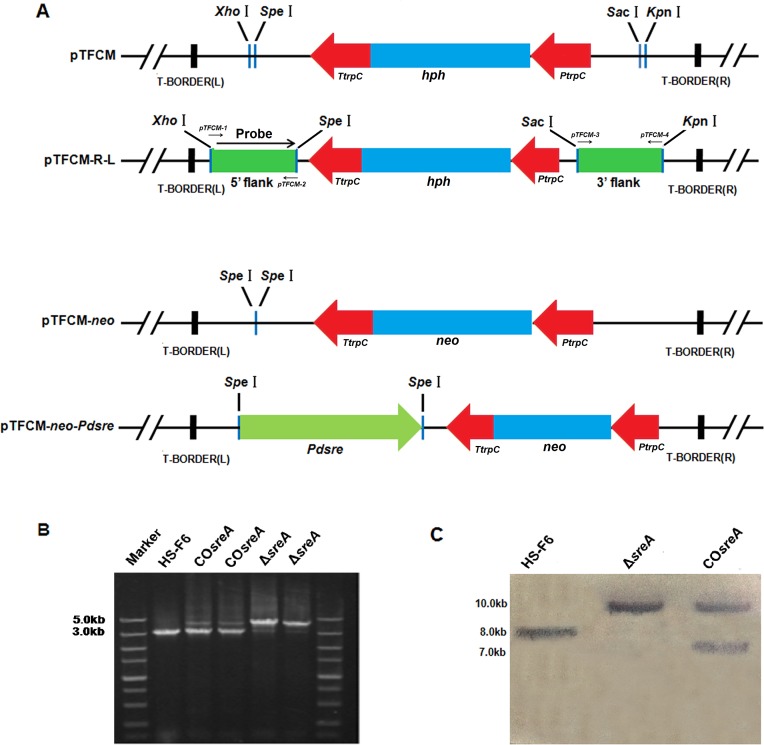
Construction and analysis of *sreA* strains. **A**: Construction of pTFCM-R-L and pTFCM-*neo*-*sreA* plasmids. B: PCR analysis of Δ*sreA*, and CO*sreA* transformants using primers *sreA*-F and *sreA*-R (**Table 1**). C: Southern blot analysis of *P*. *digitatum* wild-type PdHS-F6 and Δ*sreA*, CO*sreA* strains using a probe specific to the 5’ region of *Pdsre*. 30μg genomic DNA was digested with *Hind*III and detected using a probe specific to the 5’ region of *sreA* gene.

To analyze the function of SreA in *P*. *digitatum*, *sreA* disruption strains (Δ*sreA*) of HS-F6 (PdHS-F6m) were constructed (**Fig. 2A**). Twenty putative mutants were selected on PDA medium supplemented with 50 μg/ml hygromycin B and screened by PCR using primers *sreA-1* and *sreA-4* (**Table 1**). The 3.0-kb fragment of *sreA* in HS-F6 was replaced by a 4.0-kb fragment (the *sreA* gene disrupted with the *hph* gene) in the recombinant strains (Δ*sreA*) (as shown in **Fig. 2B**). Southern blot analysis further confirmed that a single copy was integrated. Only one strain was chosen and used for further study (**Fig. 2C**).

Twenty complementation strains (CO*sreA*) were obtained using G418 as selective marker. The insertion of *sreA* in the CO*sreA* strains was confirmed by PCR using primers *sreA-1* and *sreA-4*. The occurrence of a 3.0-kb band (*sreA*) and a 4.0-kb band (*sreA* disrupted with the *hph*) indicated the successful integration of the *sreA* gene into the genome of the Δ*sreA* strains (**Fig. 2B**). Southern blotting confirmed that the Δ*sreA* and CO*sreA* strains were successfully constructed and that a single copy of *sreA* was integrated into the genome of the Δ*sreA* strain to generate the CO*sreA* strain (**Fig. 2C**).

### Deletion of *sreA* renders *P*. *digitatum* more susceptible to triazole drug prochloraz

**Fig 3:**
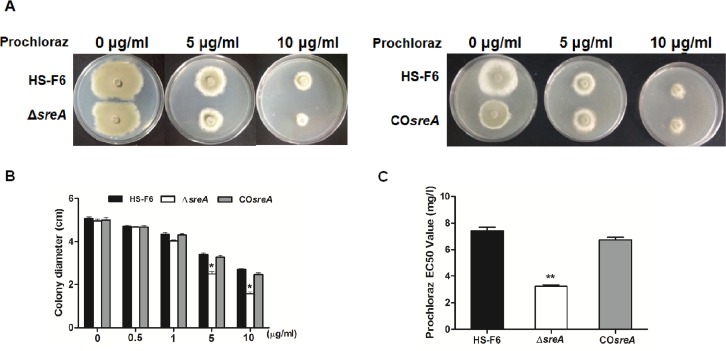
The sensitivity of *P*. *digitatum* wild-type (PdHS-F6), Δ*sreA*, and CO*sreA* strains to prochloraz. **A**: Growth assay of *P*. *digitatum* HS-F6 wild-type, Δ*sreA* and CO*sreA* strains on PDA plates with or without prochloraz (concentrations: 0, 5 and 10μg/ml). All strains were cultured at 25°C for three days. **B**: Bars represent the average diameter plus standard errors of colonies grown on PDA plates supplemented with different concentrations of prochloraz (concentrations: 0, 0.5, 1, 5,10μg/ml). **C**: Comparison of prochloraz EC_50_ values of the PdHS-F6 wild-type, Δ*Pdsre*, and CO*Pdsre* strains. Each bar represents the EC_50_ value plus standard error of three measurements. (*P<0.05; **P<0.01)

As stated above, PdHS-F6 is highly-resistant to triazole drug prochloraz and has an EC_50_ value of 7.896 mg/l, which is more than 800 times higher than prochloraz-susceptible strains. As shown in **Fig. 3**, PdHS-F6 and Δ*sreA* strains showed similar growth on the PDA plate without prochloraz, the diameters of colonies were not significantly different. However, the diameters of colonies of Δ*sreA* strains on PDA plates supplemented with prochloraz (5 μg/ml, 10 μg/ml) were smaller than those of HS-F6. After being cultured on PDA plates supplemented with different concentration of prochloraz (0, 0.5, 1, 5, 10 μg/ml) for 6 days, the diameters of the colonies were measured (**Fig. 3B**), and the EC_50_ value was calculated. The average EC_50_ value of prochloraz for the Δ*sreA* strain was 3.2 mg/l, which was less than half of the value of HS-F6 (**Fig. 3C**). However, complementation of *sreA* (PdHS-F6c) restored the EC_50_ value of the Δ*sreA* strain (average EC_50_ value of prochloraz for the CO*sreA* strain was 6.7 mg/l). These results demonstrated that the deletion of *sreA* renders *P*. *digitatum* more susceptible to triazole drug prochloraz, suggesting that *sreA* plays an important role in the resistance of *P*. *digitatum* to triazole drugs.

### Gene *sreA* is required for full virulence in *P*. *digitatum*

**Fig 4:**
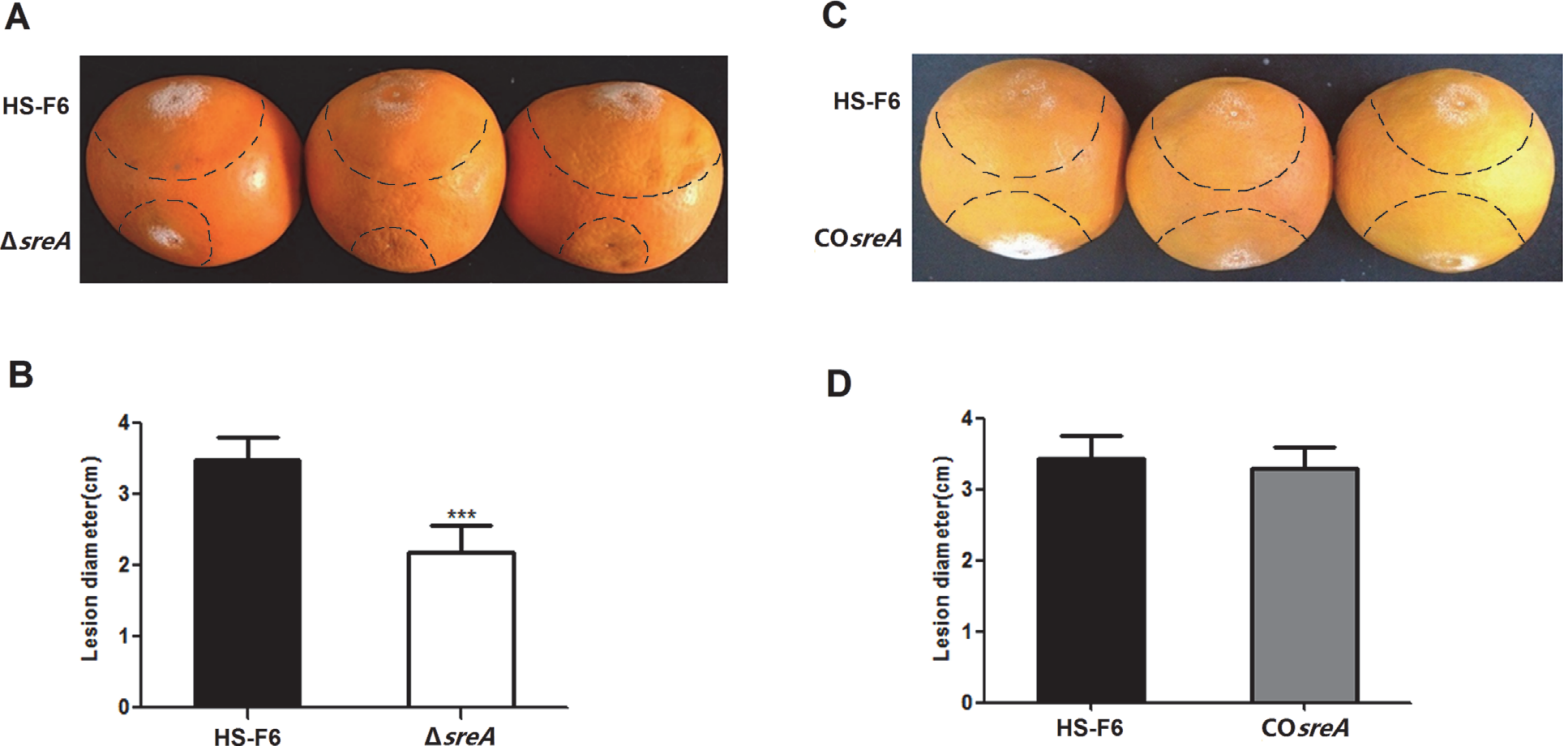
Virulence assay for the *P*. *digitatum* HS-F6, the Δ*sreA* strian, and the CO*sreA* strain. A: A 3μL aliquot of a conidial suspension (10^6^ spores ml^1^) of the HS-F6 or Δ*sreA* strain was injected into citrus fruits and incubated at 25°C for three days. B: Bars represent the mean diameter plus standard errors of 20 disease spots. (***P<0.001); **C**: 3μl conidial suspension (10^6^ spores ml^-1^) of HS-F6 or CO*sreA* strains was injected into the citrus fruits and incubated at 25°C for three days; **D**: Bars represent the mean diameter plus standard errors of 20 disease spots.

To determine whether *sreA* plays a role in the virulence of *P*. *digitatum* toward citrus fruits, a virulence assay was performed directly on these fruits. The assay results demonstrated that the symptoms in the fruits incubated with conidial suspension of the Δ*sreA* strains developed more slowly than in the fruits incubated with the wild-type conidial suspension. The mean diameter of the macerated lesions of the fruits incubated with the Δ*sreA* conidial suspension was approximately 1.90 cm at 3 days post inoculation, whereas the mean macerated lesion diameter of the fruits incubated with the HS-F6 conidial suspension was about 3.4 cm (**Fig. 4A, B**). The virulence assay results revealed that the deletion of *sreA* rendered the Δ*sreA* strain less virulent compared with the wild-type HS-F6. A further experiment was performed to confirm this result. The virulence assay demonstrated that average diameter of the macerated lesions induced by CO*sreA* was comparable to that of wild type HS-F6 (3.2 cm) (**Fig. 4C, D**). These results indicated that SreA is required for full virulence in *P*. *digitatum*.

### Transcriptional abundance of the *cyp51* genes is significantly decreased in the Δ*sreA* strain

**Fig 5:**
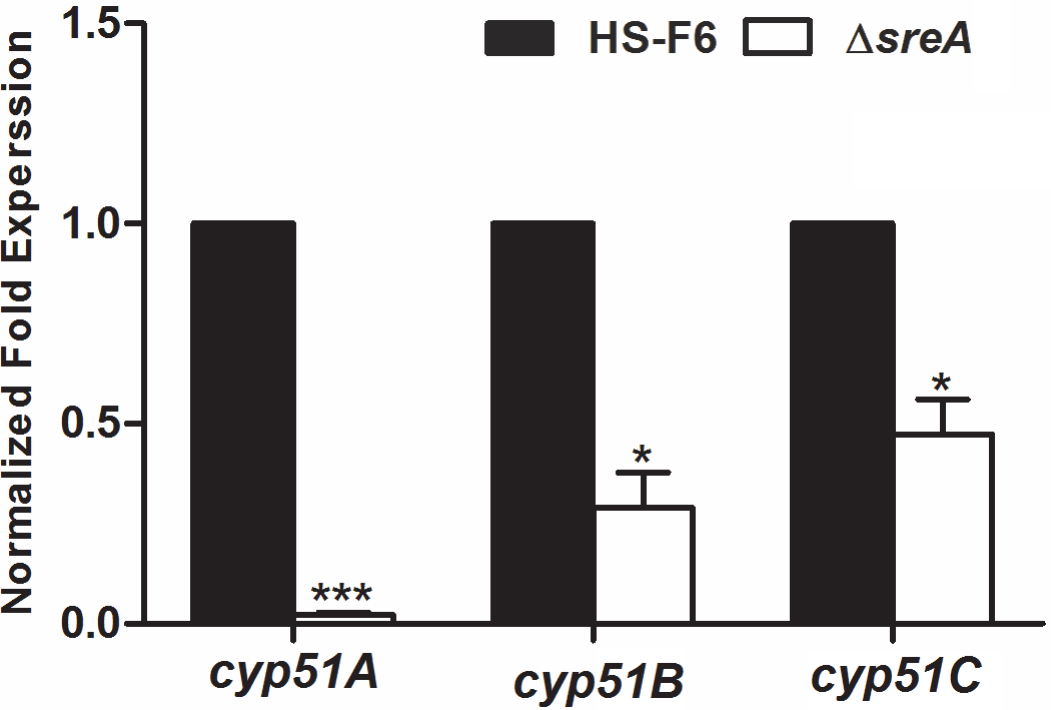
Differential *cyp51A*, *B*, and *C* transcriptional abundance for *P*. *digitatum* wild-type (HS-F6) and Δ*sreA* strains. RNA was isolated from three-day-old mycelium of HS-F6 and Δ*sreA* strains for qRT-PCR analysis. The mRNA abundance was normalized by the housekeeping gene *β-actin*. The relative expression levels were calculated using the 2^-ΔΔCt^ method. Three biological replicates were performed. (*P<0.05; ***P<0.001)

Because SrbA regulates the expression of *erg11A* gene in *A*. *fumigatus* [21], we hypothesized that its ortholog, SreA, plays a similar role in *P*. *digitatum*. To test this hypothesis, the mRNA abundance of the *cyp51* genes in PdHS-F6 wild-type and the Δ*sreA* strain was analyzed using qRT-PCR (**Fig. 5**). The results showed that transcriptional abundance of the three *cyp51* genes were all decreased in the Δ*sreA* strain, especially with regard to *cyp51A*. The normalized expression values of *cyp51A*, *cyp51B*, *and cyp51C* in the Δ*sreA* strain were 0.02, 0.29 and 0.47, respectively.

### SreA is required for transcriptional response to prochloraz in *P*. *digitatum*

**Fig 6:**
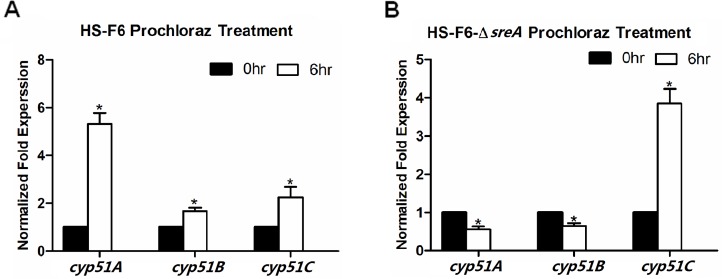
Transcriptional abundance of *cyp51* genes in HS-F6 wild-type and Δ*sre* strains after prochloraz treatment. The wild-type and Δ*sre* strain were treated with or not with prochloraz (7μg/ml) and shaking at 25°C for 6 hours. Total RNA was isolated for qRT-PCR as described in Materials and methods. **A**: Bars represent the relative expression levels of CYP51 genes in HS-F6. **B**: Bars represent the relative expression levels of CYP51 genes in the Δ*sreA* strain; mRNA abundance was normalized by the housekeeping gene *β-actin*. The relative expression levels were calculated using the 2^-ΔΔCt^ method. Three biological replicates were performed. (*P<0.05)

To investigate the role of *sreA* in the transcriptional response of *P*. *digitatum* to prochloraz, a prochloraz induction experiment was performed with the Δ*sreA* and HS-F6 strains. Total RNA was isolated from the PdHS-F6 and Δ*sreA* strains with or without prochloraz treatment and used for qRT-PCR. In the case of wild-type HS-F6, the expression levels of the *cyp51* genes were all increased after 6 h of prochloraz treatment (**Fig. 6A**); the normalized expression values in the *cyp51A*, *cyp51B* and *cyp51C* expression levels after prochloraz treatment were 5.3, 1.8, and 2.2, respectively. However, the induction of *cyp51A* and *cyp51B* by prochloraz was abolished in the Δ*sreA* strain. As shown in **Fig. 6B**, the normalized expression values for *cyp51A* and *cyp51B* mRNA abundances were 0.56 and 0.65, respectively. Notably, the increasing fold-change in *cyp51C* mRNA abundance after prochloraz treatment was higher in the Δ*sreA* strain than in the wild-type strain HS-F6 (**Fig. 6A, B**), which might be due to regulation by other transcription factors that maintained *cyp51C* transcript level in response to prochloraz.

## Discussion

A number of transcription factors associated with sterol metabolism and drug resistance have been well characterized in different clinical fungi. Upc2, a typical zinc cluster protein identified in *S*. *cerevisiae* and *C*. *albicans*, was the first documented transcription factor highly correlated with fungal drug resistance [34–37]. Deletion of *CaUpc2* rendered *C*. *albicans* increased susceptibility to a range of common antifungal drugs used in clinical therapy, and this increased susceptibility is observed for drugs targeting the ergosterol biosynthesis pathway [16]. A previous report also provided direct evidence for Upc2 in transcriptional regulation of *C*. *albicans erg* genes involved in ergosterol biosynthesis, including *erg*7, *erg11* and *erg25* [38]. The *erg11* overexpression in the Upc2 gain-of-function mutants led to strain resistance to azole fungicides [39]. SREBPs, functionally conserved in fungal kingdom, have been revealed to regulate sterol synthesis in fission yeast *S*. *pombe* as well as a number of fungal pathogens particularly grown under hypoxia conditions [20, 40]. Sre1 undergoes sterol-dependent proteolytic activation and regulates genes required for maintaining cellular sterol homeostasis. Sre1 is also required for many other fungi to regulate sterol biosynthesis, and SREBP is required for mammalian cells to regulate cholesterol and fatty acid anabolism [41–42]. SrbA from the SREBP super family is associated with hypoxia, cell polarity, full virulence, and ergosterol homeostasis in *A*. *fumigatus*. It is notable that SrbA plays a critical role for triazole drug interactions in *A*. *fumigates*, which may have clinical importance [23–24]. In most eukaryotes, including the majority of fungi, expression of sterol biosynthesis genes is regulated by SREBPs. However, in yeasts such as *S*. *cerevisiae* and *C*. *albicans*, sterol synthesis is regulated by Upc2 instead [43]. Even though Upc2 functions similarly to SrbA, they are totally different from each other in terms of the protein structure: Upc2 is a zinc finger transcription factor with a typical Gal4-type zinc finger, while SREBPs contain a bHLH domain and a characteristic tyrosine residue.

In the present study, we characterized an SREBP transcriptional factor, SreA, in *P*. *digitatum* as an ortholog of SrbA that is associated with triazole susceptibility, full virulence, and regulation of *cyp51* genes [24]. Our results demonstrated that SreA in *P*. *digitatum* may play a role similar to SrbA. The *sreA* deletion strains of *P*. *digitatum* (Δ*sreA*) were generated using *A*. *tumefaciens*-mediated genetic transformation based on the prochloraz-resistant *P*. *digitatum* strain PdHS-F6. Prochloraz is a triazole fungicide that is widely used in Europe, Australia, Asia and South America within gardening and agriculture [25]. However, with the extensive and excessive use of prochloraz, drug-resistant fungi have appeared. As reported in 2013, 78 strains of *P*. *digitatum* were isolated from citrus fruits collected in Hubei Province and 25 isolates were identified to be prochloraz-resistant, with a proportion as high as 32% [26]. For reducing the increasing economic loss due to fungicide resistance, understanding the molecular mechanisms of resistance is of practical significance.

Our results in *P*. *digitatum* demonstrated that the deletion of *sreA* increases susceptibility of PdHS-F6 to prochloraz. As shown in **Fig. 3**, the prochloraz EC_50_ value of the Δ*sreA* strain was significantly lower than HS-F6. The growth assay on PDA plates revealed that the *ΔsreA* strain grew as well as PdHS-F6 without prochloraz. However, the growth rate of Δ*sreA* strain on PDA plates supplemented with prochloraz (5 μg/ml, 10 μg/ml) was much slower than that of the HS-F6 (**Fig. 3A, B**), revealing that drug response was altered in the Δ*sreA* strain. CYP51 proteins are the targets of triazole fungicides, and the susceptibility of fungi to triazole drugs is closely related with the mutations in or the expression level of *cyp51* genes [44]. To confirm the role of SreA in the regulation of *cyp51* gene expression, the transcriptional abundance of *cyp51* genes in HS-F6 and Δ*sreA* strain were analyzed by qRT-PCR. The results revealed that the expression level of the three *cyp51* genes was decreased in the Δ*sreA* strain, particularly *cyp51A*, which almost could not be detected (**Fig. 6**). Our study thus demonstrated that SreA is an important regulator of *cyp51* genes: the deletion of *sreA* reduced the expression levels of *cyp51* genes, which are the targets of trizole fungicide prochloraz, rendering the strain more susceptible to the drug. However, SreA is not the only transcription factor regulating the expression of *cyp51* genes. In the Δ*sreA* strain, other regulators may control the expression of *cyp51* genes in response to the drug and to some extent may compensate the loss of *sreA*. In support of this notion, the EC_50_ value of the Δ*sreA* strain was not as low as the prochloraz-susceptible strain, and the Δ*sreA* strain could still grow on PDA plates supplemented with prochloraz at a relatively high concentration; nevertheless, the growth rate was much slower than that of HS-F6.

To determine whether *sreA* is associated with pathogenicity of *P*. *digitatum*, virulence assays were performed directly on citrus fruits using the HS-F6 wild-type and mutant strains. The results demonstrated that the Δ*sreA* strain was less virulent than the wild type. The symptoms in fruits incubated with a Δ*sreA* conidial suspension developed more slowly and the mean diameter of the macerated lesions was almost half of those produced by the HS-F6 wild-type strain (**Fig. 4**). As expected, the CO*sreA* strain displayed symptoms similar to the wild type, indicating that the complementation of *sreA* restored the virulence of Δ*sreA* strain. Therefore, we can conclude that SreA is required for full virulence in *P*. *digitatum*.

Fungi have evolved sophisticated mechanisms to cope with environmental stresses, including antifungal drugs, and respond to triazole drugs by altering the expression levels of effector genes. DMI-resistant isolates were initially thought to exhibit fitness penalties that would preclude DMI resistance and becoming wide-spread in nature, as reported in the studies of several important fungal pathogens of different crops [45]. In the past few years, DMI-resistant strains have also been identified in human pathogenic fungi. For example, *erg25* transcription is induced in response to itraconazole treatment in *C*. *albicans* [44]. Besides, the triazole-induced overexpression of *cyp51* genes in fungi has been regarded as an important fungal self-protective mechanism towards a range of fungicides [24]. In this study, we compared the *cyp51* mRNA expression in PdHS-F6 and Δ*sreA* strains before and after prochloraz treatments. In the HS-F6 wild-type strain, the expression level of *cyp51* genes were all increased after prochloraz induction for 6 h (**Fig. 6A**). However, the increase in *cyp51* expression levels, except *cyp51C*, was abolished in the Δ*sreA* strain. The further increased mRNA abundance of *cyp51C* in response to prochloraz-treatment in the Δ*sreA* strain might be attributed to the regulating by other transcription factors in response to the drug. However, the relatively decreased expression level of *cyp51C* in the Δ*sreA* strain demonstrated that *sreA* could regulate the expression of *cyp51C* to some extent. These results indicated that SreA is an important regulator of *cyp51* genes. The deletion of *sreA* renders *P*. *digitatum* unable to cope with prochloraz properly, making it more susceptible to the antifungal drug.

With the widespread and constant use of triazloe antifungal drugs, resistance has been selected in many fungal species. Most of this resistance is due to the mutation and overexpression of target *cyp51* genes or the mutation of their regulatory genes [5–6, 18–19]. Besides, the overexpression of transporter-encoding genes also contributed to fungi resistance [46]. The documented alterations in transcription factors, particularly in their DNA binding sites, could affect drug resistance in the pathogenic yeast *C*. *albicans*, tumorigenesis in hosts, and for resistance of the malarial parasite *Plasmodium vivax* [18, 47–51]. Therefore, identifying new target genes and the designing of new drugs is of great significance to control the spread of resistant fungi. Previously, we reported the cloning, expression, and characterization of *cyp51* from *P*. *digitatum* and *Ustilago maydis* [52–55]. The structural characteristics of the interaction between heterologous CYP51 and commercial azoles were also analyzed by binding assays. A series of new 2-azolyl-3, 4-dihydroquinazolines 6 was synthesized by the direct cyclization of imidazole or 1, 2, 4-triazole with carbodiimides 4, and the preliminary bioassay results demonstrated that these compounds exhibited well to significant fungicidal activity against *P*. *digitatum* [54]. The screening of new DMI fungicides based on optimized expression has been carried out for the first time in *Ustilago maydis* [55]. Recently, a cell-based high-throughput screen has identified small-molecule inhibitors of the Upc2-dependent induction of sterol gene expression in response to azole drug treatment. The compounds were growth-inhibitory and could attenuate antifungal-induced sterol gene expression *in vivo* [56]. *Thus*, *as a regulator of cyp51*, SreA, with an essential function in *P*. *digitatum* fungicide resistance, represents a promising research direction to uncover a new fungus-specific antifungal drug targets.
